# Electromagnetic Brain Stimulation in Patients With Disorders of Consciousness

**DOI:** 10.3389/fnins.2019.00223

**Published:** 2019-03-18

**Authors:** Pierre Bourdillon, Bertrand Hermann, Jacobo D. Sitt, Lionel Naccache

**Affiliations:** ^1^Department of Neurosurgery, Adolphe de Rothschild Foundation, Paris, France; ^2^Sorbonne Université, Faculté de Médecine Pitié-Salpêtrière, Paris, France; ^3^Institut du Cerveau et de la Moelle Épinière, ICM, PICNIC Lab, Paris, France; ^4^Inserm U 1127, Paris, France; ^5^CNRS, UMR 7225, Paris, France; ^6^Department of Neurology, Neuro ICU, Groupe Hospitalier Pitié-Salpêtrière, AP-HP, Paris, France; ^7^Department of Neurophysiology, Groupe Hospitalier Pitié-Salpêtrière, AP-HP, Paris, France

**Keywords:** consciousness, disorders of consciousness, deep brain stimulation, vagus nerve stimulation, transcranial magnetic stimulation, transcranial electric stimulation, transcranial direct current stimulation, transcranial alternative current stimulation

## Abstract

Severe brain injury is a common cause of coma. In some cases, despite vigilance improvement, disorders of consciousness (DoC) persist. Several states of impaired consciousness have been defined, according to whether the patient exhibits only reflexive behaviors as in the vegetative state/unresponsive wakefulness syndrome (VS/UWS) or purposeful behaviors distinct from reflexes as in the minimally conscious state (MCS). Recently, this clinical distinction has been enriched by electrophysiological and neuroimaging data resulting from a better understanding of the physiopathology of DoC. However, therapeutic options, especially pharmacological ones, remain very limited. In this context, electroceuticals, a new category of therapeutic agents which act by targeting the neural circuits with electromagnetic stimulations, started to develop in the field of DoC. We performed a systematic review of the studies evaluating therapeutics relying on the direct or indirect electro-magnetic stimulation of the brain in DoC patients. Current evidence seems to support the efficacy of deep brain stimulation (DBS) and non-invasive brain stimulation (NIBS) on consciousness in some of these patients. However, while the latter is non-invasive and well tolerated, the former is associated with potential major side effects. We propose that all chronic DoC patients should be given the possibility to benefit from NIBS, and that transcranial direct current stimulation (tDCS) should be preferred over repetitive transcranial magnetic stimulation (rTMS), based on the literature and its simple use. Surgical techniques less invasive than DBS, such as vagus nerve stimulation (VNS) might represent a good compromise between efficacy and invasiveness but still need to be further investigated.

## Introduction

Loss of consciousness and arousal are frequent after severe brain injuries. Usually, patients recover from this transient state of coma to a normal state of consciousness even though they can suffer from various cognitive deficits. However, in some cases, despite vigilance improvement, disorders of consciousness (DoC) persist. Several states of impaired consciousness have thus been defined, according to whether the patient exhibits only reflexive behaviors as in the vegetative state/unresponsive wakefulness syndrome (VS/UWS) or purposeful behaviors distinct from reflexes as in the minimally conscious state (MCS) (Giacino et al., 2002). This latter category has been recently refined to distinguish MCS ‘minus’ (MCS-) patients from MCS ‘plus’ patients (MCS+) according to the absence/presence of command following and/or intelligible verbalizations (Bruno et al., 2011). While this MCS label leaves open the issue of conscious state in these patients, it indicates with certitude that, unlike in VS/UWS, cortical networks contribute overtly to the behavior. In other terms, MCS can be reinterpreted as a cortically mediated state (CMS), more prone to evolve to recovery of consciousness than VS/UWS (Naccache, 2018). According to current classifications, a patient emerges from MCS (exit-MCS or EMCS) whenever he is able to communicate or make functional use of objects. Importantly, DoC must be differentiated from the locked-in syndrome (LIS) in which patients are conscious but lack the ability to communicate due to a disruption of motor tracts in the brainstem.

The current gold standard to diagnose these states of consciousness is the behavioral examination using a dedicated scale, the Coma Recovery Scale - revised (Kalmar and Giacino, 2005). However, recent studies have shown that a wilful modulation of brain activity could be detected in some clinically unresponsive patients (Owen et al., 2006; Edlow et al., 2017), a situation referred to as cognitive-motor dissociation (CMD). This new concept has brought the need of new classifications integrating active and passive brain-imaging to tract purposeful behaviors/consciousness (Engemann et al., 2018).

In parallel, several theories of consciousness have been developed. While some authors postulate than consciousness stem from a brain-scale cortico-cortical communication (global workspace theory; Dehaene et al., 2006; Dehaene and Changeux, 2011), others claim that consciousness arises from the coordinated activity within thalamo-cortical as well as non-thalamic ascending reticular activating system (ARAS) pathways (Edlow et al., 2012; Jang and Kwon, 2015; Jang et al., 2018), or from fronto-pallido-thalamo-cortical loops (meso-circuit hypothesis, Schiff, 2010). According to all of these theories, the common feature in DoC pathophysiology would be the disruption of a complex and organized high-order activity among large-scale neural networks.

**FIGURE 1:**
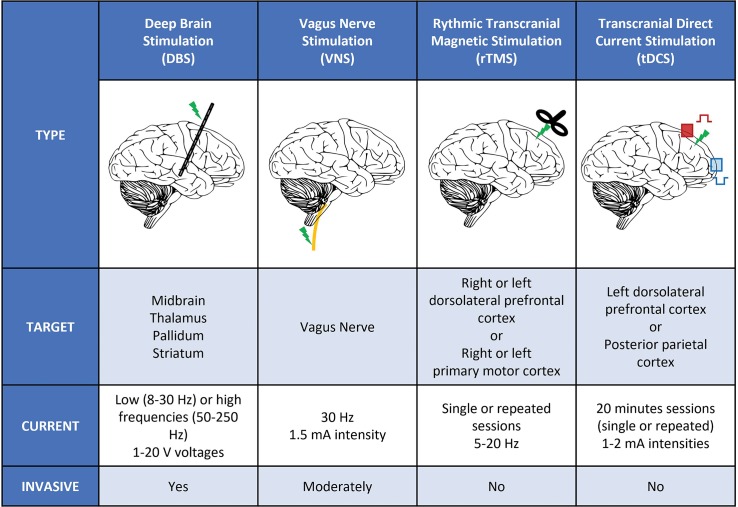
Different types of stimulation used in DoC patients. Schematic representation of the different types of invasive a non-invasive stimulation used in DoC patients. We listed the main targets and stimulation parameters (intensities, voltages, frequencies, and number of sessions) used in clinical studies. DBS, deep brain stimulation; Hz, Hertz; mA, milli-ampere; rTMS, rhythmic transcranial magnetic stimulation; tDCS, transcranial direct current stimulation; V, Volt; VNS, vagus nerve stimulation.

In spite of these progresses in our understanding of DoC pathophysiology, efficient therapeutics is still lacking. Except for the moderate acceleration of recovery of traumatic brain injury (TBI) with amantadine (Giacino et al., 2012) and the rare and transient paradoxical effect of zolpidem (Whyte and Myers, 2009; Whyte et al., 2014), neuropharmacological therapies are disappointing and, most of the time, neuro-rehabilitation, despite a limited impact (Giacino et al., 2013), is the only treatment. Within this context, ‘electroceuticals,’ relying on the direct or indirect electro-magnetic stimulation of the brain, may be promising tools to restore consciousness in DoC patients (Figure 1). We conducted a narrative review of the use of these techniques in DoC patients by conducting a Pubmed/MEDLINE literature search up to December 2018 with the terms: ‘disorders of consciousness,’ ‘consciousness’ AND ‘non-invasive brain stimulation stimulation,’ ‘transcranial electrical stimulation,’ ‘transcranial direct current stimulation,’ ‘transcranial alternative current stimulation,’ ‘transcranial random noise stimulation,’ ‘transcranial magnetic stimulation,’ ‘invasive brain stimulation,’ ‘deep brain stimulation.’ We selected original papers with patients data based on their importance in the field.

## Invasive Electric Stimulation

### Deep Brain Stimulation

Stereotactic surgical methodology was first described in the late 19th century (Apra et al., 2016), but applications in neurological diseases on the basis of neurophysiological principles started only in the second half of the 20th century (Giller et al., 2017; Bourdillon et al., 2018). Performing a lesion on deep mesencephalic or diencephalic small structures with wide projections on large cortical areas was exciting perspectives in psychiatric and neurological fields and drastically reduced the morbidity of the surgical procedures (Miocinovic et al., 2013; Bourdillon et al., 2017). These lesional procedures were indicated in pathologies producing positive signs (like tremor or dystonia) but were useless in pathologies in which negative signs were preponderant, such as disorder of consciousness (DoC). In this context, electric stimulation in human patients by means of stereotactically placed intracranial deep electrodes was developed. DoC, which was then considered as a default of cortical activation consecutive to an interruption of the projections of the ARAS through the diencephalon to the cortex, was indeed one of the first pathologies in which deep brain stimulation (DBS) was used (McLardy et al., 1968; Hassler et al., 1969). Despite an exciting effect of these first reports of pallidal and thalamic stimulation on the arousal of vegetative patient, no other study was done until the DBS was democratized in the late 1980’s by its use in Parkinson disease (Benabid et al., 1987).

#### Patients and Clinical Response

Since 1968, a systematic review of the literature (through Medline, Embase, and web of Science) found that ten studies reporting 78 unique DoC patients who underwent DBS have been published (Table 1) (McLardy et al., 1968; Tsubokawa et al., 1990; Cohadon and Richer, 1993; Schiff et al., 2007; Yamamoto et al., 2010; Wojtecki et al., 2014; Adams et al., 2016; Magrassi et al., 2016; Chudy et al., 2018; Lemaire et al., 2018).

**Table 1:** Deep brain stimulation studies in DoC patients.

Study	Design/Control	Population	Target/Stimulation parameters	Behavioral effects	Electrophysiological/metabolic effects	Side effects
McLardy et al., 1968	Case report/None	1 (considered as) VS/UWS	Left thalamus; midbrain (intralaminar nuclei/reticular formation) / 250Hz, 1ms	No modifications of consciousness, left hand spontaneous movement	No post procedure electrophysiological nor metabolic evaluation available	None
Hassler et al., 1969	Case report/None	1 (considered as) VS/UWS	Left ventral anterior thalamus; right pallidum / Left, 25-30Hz, 20V, 1-3ms; Right 8Hz, 30V, 1-3ms	“Improvement” of consciousness, vocalizations, left limbs spontaneous movement	EEG recordings showed a disappearance of a unilateral delta focus which is replaced by an alpha activity	None
Tsubokawa et al., 1990	Open-label/None	8 patients (VS/UWS)	Central thalamic nuclei; nucleus cuneiformis (reticular formation)/50 Hz, 0–10 V	4 recoveries (PCS 2–4 = > 8-9) 1 responder (PCS 2–4 = > 7) 3 failures (PCS 2–4 = > 3-5)	Increase of spectral power and desynchronization on EEG in the 4 patients who recovered/Increase on the brain perfusion on MRI in these patients	None
Cohadon and Richer, 1993	Open-label/None	25 patients (VS/UWS)	Central nucleus of the thalamus/50 Hz, 5–10 V, 5 ms	1 moderate disabilities (GOS) 10 severe disabilities (GOS) 12 no effect *(2 patients died before the endpoint)*	No post procedure electrophysiological nor metabolic evaluation available	2 died (unrelated to surgical procedure)
Schiff et al., 2007	Case report, Cross-over RCT/Sham	1 MCS	Anterior intralaminar thalamic nuclei / 100Hz, 4V	Fluctuant increase in CRS-R subscales, better feeding and motor behaviors, restoration of communication	No post procedure electrophysiological nor metabolic evaluation available	None
Yamamoto et al., 2010 *(includes publications since 2002)*	Open-label/None	21 patients (VS/UWS)	Centro-median nucleus of the thalamus; midbrain (reticular formation) / 25Hz, various intensities	8 became MCS or EMCS 13 remain VS/UWS	The 8 patients who recovered from VS showed desynchronization on continuous EEG frequency analysis/Increase on the brain perfusion on MRI in these patients	None
Wojtecki et al., 2014	Case report/None	1 MCS	Internal medullary lamina; nuclei reticularis thalami/70–250 Hz, various intensities	No modifications of consciousness	Modulation of oscillatory activity in the beta and theta band within the central thalamus accompanied by an increase in thalamocortical coherence in the theta band	None
Magrassi et al., 2016	Open-label/None	3 patients (1 MCS, 2 VS/UWS)	Anterior intralaminar nuclei; paralaminar Areas/80–110 Hz, various intensities	Increase of CRS-R in all of the 3 patients: 14 = > c15 8 = > 11 6 = > 9	Increase of theta and gamma power spectrum in EEG after 1 month of stimulation. No modifications of the evoked potentials.	1 postoperative intraparenchymal hematoma
Adams et al., 2016	Case report/None	1 MCS	Anterior intralaminar thalamic nuclei/100 Hz, 4 V	Variable increase of CRS-R (11–14)	Long term re-emergence of sleep patterns	None
Chudy et al., 2018	Open-label/None	14 patients (4 MCS, 10 VS/UWS)	Central thalamic nuclei / 25 Hz, 2.5–3.5 V, 90 μs	3 MCS became EMCS; 1 VS became MCS; 7 had no improvement of consciousness *(3 patients died before the endpoint)*	No post procedure electrophysiological nor metabolic evaluation available	3 died (unrelated to surgical procedure)
Lemaire et al., 2018	Cross-over RCT/Sham	5 patients (4 MCS, 1 VS/UWS)	Dual pallido-thalamic / 30-Hz, 6V, 60μs	1 VS/UWS and 1 MCS had an significant improvement of the CRS-R.	The metabolism of the medial cortices increased specifically in the two responders	1 postoperative bronchopulmonary infection

*CRS-R, Coma Recovery Scale – Revised; DoC, disorders of consciousness; EEG, electroencephalogram; EMCS, Emergence from Minimally Conscious State; GOS, Glasgow Outcome Scale; MCS, Minimally Conscious State; PCS, Prolonged Coma Scale; RCT, randomized controlled trial; VS/UWS, vegetative state/unresponsive wakefulness syndrome.*

A response was noticed in 30 of the 67 patients classified as VS/UWS and in 6 of the 11 MCS. The definition of “response” is highly variable throughout the studies as the outcome measures have dramatically evolved since the 1970’s. Nevertheless, the clinical description provided in the oldest studies are all compatible with an improvement on the Coma Recovery Scale revised (CRS-R), the outcome measure systematically used nowadays.

Etiologies of DoC were traumatic brain injuries (27 patients), anoxic causes (12 patients) and vascular causes (13 patients) but were not reported in the largest series (Cohadon and Richer, 1993). Throughout the literature, it is unclear whether etiology is an outcome predictive factor (Vanhoecke and Hariz, 2017). Severe side effects may occurs during DBS. The safety is reported in Table 1.

It is worth mentioning that two studies, totalizing 5 patients, were not taken into account as the included patients did not fit with the present definition of DoC patients (Sturm et al., 1979; Hosobuchi and Yingling, 1993).

#### Targets and Parameters of Stimulation

Multiple targets have been tested including the reticular formation (McLardy et al., 1968; Tsubokawa et al., 1990; Yamamoto et al., 2010), the central nucleus of the thalamus (McLardy et al., 1968; Tsubokawa et al., 1990; Cohadon and Richer, 1993; Schiff et al., 2007; Yamamoto et al., 2010; Wojtecki et al., 2014; Adams et al., 2016; Chudy et al., 2018), the anterior intralaminar nuclei and paralaminar areas (Magrassi et al., 2016). In two studies, pallidal stimulation was associated to thalamic targets (Hassler et al., 1969; Lemaire et al., 2018). The multiplicity of targets in the limited number of both VS/UWS and MCS patients makes it impossible to identify the superiority of a procedure among the others. However, all these targets correspond anatomically to the projections of the ARAS through the thalamus to the cortex. Consequently, despite an apparent heterogenicity of the DBS targets, all the published studies report observations of the modulation of the same pathway making the interpretation of the overall results easier. Low-frequency stimulation (up to 50 Hz) was mostly used (Hassler et al., 1969; Tsubokawa et al., 1990; Cohadon and Richer, 1993; Yamamoto et al., 2010; Chudy et al., 2018), but some studies reported results using high frequency stimulations (up to 100 Hz) (Schiff et al., 2007; Wojtecki et al., 2014; Adams et al., 2016; Magrassi et al., 2016). The impact of the parameters of stimulation on the clinical response remains unclear (Kundu et al., 2018).

#### Limitations

One of the most important criticisms on the published studies is about the time frame. The Multi Society Task Force on persistent VS/UWS has published that spontaneous recovery from non-anoxic VS/UWS lasting longer than 1 month occurs in 30% of patients at 6 months and in 43% at 12 months (Multi-Society Task Force on PVS, 1994; Vanhoecke and Hariz, 2017). This observation is not limited to VS as 83% of the patients emerged from MCS after 6 months (Lammi et al., 2005). Yet, most studies report DBS performed within the year following the brain injury (Hassler et al., 1969; Tsubokawa et al., 1990; Cohadon and Richer, 1993; Yamamoto et al., 2010; Chudy et al., 2018) so that, in the 29 out of the 41 patients who improved after DBS, spontaneous recovery cannot be excluded.

Another limitation is the selection of the patients on clinical criteria. Very different lesions in the central nervous system can lead to the same clinical presentation. For instance, VS/UWS may result from diffuse cortical lesions as well as from a very focal lesion in the brainstem of the ARAS. In the first situation, DBS will modulate a damaged cortex with altered capacity of long distance synchronization while, in the second, a modulation of the thalamus will have an effect on a preserved cortex. The most recent studies tend to take this into account by excluding anoxic causes (Lemaire et al., 2018) or trying to identify the potential connectivity that the DBS may restore (Schiff et al., 2007; Magrassi et al., 2016). Nonetheless, most of the studies mixed patients with similar clinical presentations but with a potentially great physio-pathological heterogenicity.

#### Perspectives

To avoid the methodological issues due to the study design of the initial studies, DBS should not be offered within the interval of 1 year of possible spontaneous recovery from DoC (Vanhoecke and Hariz, 2017). The double-blind design introduced in DBS for DoC by Schiff (Schiff et al., 2007) should lead to less biased clinical conclusions and to exclusion of spontaneous recovery.

To overcome the heterogenicity of the patients in terms of physiopathology and to choose the most appropriate target for a single patient, an option could be to take advantage of the recent advances in the description of the physiology and anatomy of DoC patients. The structural integrity of the white matter tracts (Weng et al., 2017; Zheng et al., 2017; Velly et al., 2018; Wang et al., 2018; Wu et al., 2018) and the functional connectivity assessed by electrophysiology (Bekinschtein et al., 2009; King et al., 2013; Sitt et al., 2014; El Karoui et al., 2015) or MRI (Owen et al., 2006; Cruse et al., 2011; Boly et al., 2012; Laureys and Schiff, 2012; Casali et al., 2013) are becoming routine practice in DoC patients evaluation so that patient level connectivity maps tend to become available. Definition of a minimal criterion of brain connectivity before trailing with DBS could be an interesting option to appropriately select patients in whom DBS may be beneficial. Moreover, DBS target could be personalized, in such selected patients, to restore long range connectivity in low frequency band through deep nuclei or tracts considered as damaged nodes in the network. Finally, DBS could be proposed in priority to patients in a CMS (Naccache, 2018) defined by the existence of substantial cortical functional networks revealed by behavioral examination (e.g., MCS patient and in particular MCS+ patients and/or by functional brain-imaging (including CMD patients). Indeed, such patients are predicted in theory to benefit the most from sub-cortical activation of residual cortical networks.

### Vagus Nerve Stimulation

More recently, as a less invasive alternative to DBS, vagus nerve stimulation (VNS) has been tested in a DoC patient (Corazzol et al., 2017). The vagus nerve directly modulates activity in the brainstem and, through the nucleus of the solitary tract, reaches the dorsal raphe nuclei and the thalamus (Rutecki, 1990). Its positive effect on reticular formation, thalamus and forebrain metabolism has been established (Henry et al., 1999). In addition to this modulation of the ARAS, very similar to what is observed in DBS, VNS is known to enhance the releasing of norepinephrine in the thalamus through an enhancement of the neuronal firing of the locus coeruleus, a crucial pathway for arousal (Dorr and Debonnel, 2006).

The unique patient reported with this technique was a 35 years old man in a VS/UWS for 15 years after a severe TBI. The maximum effect was obtained with a 1 mA stimulation. The CRS-R increased, from a score of 5 at baseline to 10 and the patient was then classified as MCS. The pre and post stimulation high density EEG showed a significant increase in theta band (4–7 Hz) and the 18F-FDG PET results corroborated these findings and reveal an increase of activity in fronto-parietal and basal ganglia regions. These results are coherent with an emergence of the patient from the VS/UWS to the MCS. This observation demonstrates the ability of vagus nerve stimulation to modulate large-scale connectivity and its therapeutic potential in DoC patients.

## Non-Invasive Electric and Magnetic Stimulation

By analogy with DBS, the idea that externally applied electrical current on the scalp could be used to probe brain-behavior relationship arose around 40 years ago (Merton and Morton, 1980). However, the huge intensities used at this time (∼ 20 A) led to important side effects, and this breakthrough was not immediately pursued. Only since the end of the 1990s, non-invasive brain stimulation (NIBS) was refined and gained interest in neuroscience with the emergence of two main methods, transcranial electrical stimulation (tES) and transcranial magnetic stimulation (TMS). In the recent years, both have been proposed as therapeutic tools for various conditions, among which DoC, with the main advantage of being easier to implement and not invasive as compared to DBS and VNS. However, given the greater studies heterogeneity, their results will be presented separately.

### Transcranial Magnetic Stimulation

#### Principle

Transcranial magnetic stimulation is a non-invasive stimulation technique modifying cortical excitability through the delivery of magnetic impulses generated by the flow of high-density electric current through a magnetic coil placed over the scalp. Single or short pulses of TMS can trigger firing of action potentials and allow to interact with the underlying brain activity with a high temporal resolution with excitatory or inhibitory effect depending on the modalities. These on-line TMS properties are mainly used in neuroscience to probe the function and connections of targeted brain regions. In DoC patients, such procedures have been employed to explore motor pathways’ integrity and complexity of information processing and to index consciousness (Casali et al., 2013). Therapeutic studies rely on another type of TMS taking advantages of the neuromodulatory after-effects induced by repetitive stimulation (rTMS). These longer term effects are thought to be related to changes in synaptic plasticity by modulation of glutamatergic and GABAergic balance (Stagg et al., 2009) and non-synaptic pathways (Ardolino et al., 2005).

#### Clinical Studies

Despite several studies (Table 2), the level of evidence supporting the therapeutic use of rTMS in DoC patients is low (Lefaucheur et al., 2014). Indeed, most of them are uncontrolled trials targeting heterogeneous patients with small sample size and various stimulation protocols.

**Table 2:** Transcrania lmagnetic stimulation studies in DoC patients.

Study	Design/Control	Population	Target/Stimulation parameters	Behavioral effects	Electrophysiological effects	Side effects
Louise-Bender Pape et al., 2009	Case report/None	1 VS/UWS patient	Right DLPFC/30 sessions over 6 weeks of 10 Hz rTMS (300 paired-pulse) at 110% RMT	No significant (trend) improvement of DOC Scale	Improvement of latencies of auditory brainstem evoked potentials	None
Piccione et al., 2011	Case report/Median nerve stimulation	1 MCS patient	Left M1/2 sessions of 20 Hz rTMS (10 trains of 100 stimuli) at 90% RMT	Increased CRS-R score lasting 6 h after stimulation	Increase of absolute and relative power in delta, alpha and gamma band	None
Manganotti et al., 2013	Open-label/None	6 patients (3 VS/UWS and 3 MCS)	Left or right M1/1 session of 20 Hz rTMS (10 trains of 100 stimuli) at 120% RMT	Improvement of consciousness in only 1 patient	Increase of absolute and relative power in delta, alpha and gamma band and reactivity in the responding patient	None
Pape et al., 2014	Open-label/None	2 patients	Right DLPFC/30 sessions over 6 weeks of 10 Hz rTMS (300 paired-pulse) at 110% RMT	Not assessed	Not assessed	One epileptic seizure
Xie et al., 2015	Open-label/Case-control	20 patients (2 coma, 11 VS/UWS, 7 MCS) of which 10 were stimulated	Right DLPFC/28 sessions over 28 days of 5 Hz rTMS	6 out of 10 patients stimulated showed CRS-R improvement persisting at 4 weeks	Increase of alpha power and decrease of delta power	Not reported
Naro et al., 2015a	Not randomized/Sham	10 patients (all VS/UWS) and 10 healthy controls	Right DLPFC/1 session of 10 Hz rTMS (1000 pulses) at 90% RMT	No significant group effect but small short-lasting improvement in 3 patients on the motor subscale of the CRS-R	No significant effect at the group level, but some short-lasting modulation of motor evoked potentials in the 3 responding patients	None
Cincotta et al., 2015	Cross-over RCT/Sham	11 patients (all VS/UWS)	Left M1/5 sessions over 5 days of 20 Hz rTMS (1000 pulses) at 90% RMT	No significant differences in CRS-R scores between stimulation and sham	No significant changes on EEG (Synek classification)	None
Liu et al., 2016	Cross-over RCT/Sham	10 patients (5 VS/UWS, 5 MCS)	Left M1/1 session of 20 Hz rTMS (1000 pulses) at 100% RMT	No behavioral effect	Significant changes in hemodynamic parameters (mean and peak velocity of middle cerebral artery) on transcranial doppler only in MCS	None
Bai et al., 2017	Case report/None	1 MCS patient	Left DLPFC/20 sessions over 20 days of 10 Hz rTMS (1000 pulses) at 90% RMT	Improvement of CRS-R after 20 sessions	Concomitant improvement of perturbational complexity index, global mean field power and motor evoked potential.	None
Xia et al., 2017	Prospective/Not controlled	16 patients (11 VS/UWS and 5 MCS)	Left DLPFC/20 sessions over 20 days of 10 Hz rTMS (1000 pulses) at 90% RMT	Improvement of CRS-R score in all MCS patients and 4/11 VS/UWS persisting 10 days after stimulation.	None	None
Xia et al., 2017	Prospective/Not controlled	18 patients (12 had repeated sessions for 20 days)	Left DLPFC/20 sessions over 20 days of 10 Hz rTMS (1000 pulses) at 90% RMT	Overlapping population with the previous study. No statistical testing.	Decreased low-frequency band power and increased high-frequency band power, especially in MCS	None
He et al., 2018	Cross-over RCT/Sham	6 patients (3 VS/UWS, 2 MCS and 1 EMCS)	Left M1/5 sessions over 5 days of 20 Hz rTMS (1000 pulses) at 100% RMT	No significant differences in CRS-R. One patient improved after real stimulation.	Increase delta, theta, alpha and beta power spectra in the responding patient.	Not reported
Liu et al., 2018	Cross-over RCT/Sham	7 patients (2 VS/UWS and 5 MCS)	Left M1/5 sessions over 5 days of 20 Hz rTMS (1000 pulses) at 100% RMT	No significant changes of CRS-R scores	No significant changes in functional connectivity on EEG	None

*CRS-R, Coma Recovery Scale – Revised; DLPFC, dorsolateral prefrontal cortex; DoC, disorders of consciousness; EEG, electroencephalogram; EMCS, Emergence from Minimally Conscious State; M1, primary motor cortex; MCS, Minimally Conscious State; RCT, randomized controlled trial; RMT, resting motor threshold; rTMS, repetitive transcranial magnetic stimulation; VS/UWS, vegetative state/unresponsive wakefulness syndrome.*

The first description of therapeutic TMS in DoC dates back to 2009, when Louise-Bender Pape et al. (2009) stimulated a VS patient daily for 6 weeks with 10 Hz rTMS over the right dorsolateral prefrontal cortex. While the patient presented some behavioral improvement followed by an improvement of amplitudes and latencies of brainstem auditory evoked potentials, these changes did not reach statistical significance. A second case reports an MCS patient found similar results with a transient augmentation of CRS-R score (up to 6 h) paralleled with spectral power changes on resting state EEG after two sessions of 20 Hz rTMS. This observation was latter matched by another similar case report (Bai et al., 2016).

While these first cases failed to show consistent behavioral effect on consciousness, they served as proof-of-concept supporting the safety of this procedure in DoC patients. They were thus followed by prospective open-label studies including at most 16 patients using either one session of 20 Hz stimulation over M1 (Manganotti et al., 2013), one (Naro et al., 2015b) or 30 sessions of 10 Hz rTMS over the right-DLPFC (Pape et al., 2014), or 28 sessions of 5 Hz rTMS over the same site (Xie et al., 2015). Only the latter yielded an improvement of CRS-R in 6 out of 10 patients stimulated, with a long-lasting effect persisting at 4 weeks. A more recent study by Xia et al. (2017) also seemed to show a potential benefit of DLPFC stimulation, albeit at higher frequency (10 Hz), with an increase in CRS-R scores in 5 out 5 MCS patients and 4 out of 11 VS/UWS, remaining 10 days after the end of the stimulation.

As for cross-over double-blind randomized controlled trials of rTMS in DoC, only four studies were conducted, between 2015 and 2018, with a total number of 34 patients included. They all assessed the efficacy of 20 Hz rTMS over the left M1 in comparison to a sham control condition. None of them demonstrated consciousness improvement by stimulation, regardless of whether the protocol consisted in a single session (Liu et al., 2016) or in daily sessions over 5 days (Cincotta et al., 2015; He et al., 2018; Liu et al., 2018). These studies only showed some minor EEG changes in power spectra or hemodynamic parameters monitored by transcranial doppler.

Regarding the safety of rTMS in DoC patients, these studies seemed to support its relative innocuity, even though epileptic seizures attributable to stimulation were reported in at least one subject (Louise-Bender Pape et al., 2009; Pape et al., 2014). Given the small number of patients included, this should be taken with caution, as it is known that seizures can be elicited by TMS in healthy subjects, with an increasing risk in brain-lesioned patients and with a history of seizures, two frequent conditions in DoC patients.

Although the great diversity of stimulation frequency (5, 10, 20 Hz), intensity (from 90 to 120% of resting motor threshold), site of stimulation (left or right prefrontal cortex or primary motor cortex) and number of sessions (single or repeated) makes it hard to draw definite conclusions, the few positive results demonstrating an impact of rTMS on patients’ consciousness are casting shadow over potential of rTMS in this condition. Moreover, TMS protocols are not easy to implement at bedside and require a specialized expertise and dedicated material, which questions its accessibility in the many structures (intensive care unit, neurology and rehabilitation facilities, nursing home or even at home) taking care of DoC patients. In respect to this, tES techniques are superior to TMS.

### Transcranial Electric Stimulation

#### Transcranial Direct Current Stimulation

The most used tES technique is transcranial direct current stimulation (tDCS), which delivers a continuous and weak intensity current (1–2 mA) to the scalp through a bipolar montage (the current flowing from an anode to a cathode). Although some controversies are still hanging regarding the ability of these induced electric fields to elicit clinically relevant modification of the brain activity (Vöröslakos et al., 2018), considerable evidence shows that tDCS is able to modulate the neural resting state membrane potential polarization depending on both the polarity of stimulation (Nitsche and Paulus, 2001) and of the underlying brain activity by fine tuning of synaptic gains (Lafon et al., 2017). Interestingly, as for rTMS, tDCS stimulation lasting more than a few minutes is able to induce after-effects mediated mainly by synaptic pathways [modulation of LTP and LTD (Kronberg et al., 2017) through NMDA-dependent synaptic plasticity (Liebetanz et al., 2002; Nitsche et al., 2003)] and other non-synaptic pathways (Gellner et al., 2016). Initially, tDCS was mainly targeted to probe brain functions in healthy subjects and its first therapeutic use goes back to Hummel et al. (2005). Since then, numerous studies applied tDCS in various neurologic (Parkinson’s disease, dystonia, post-stroke or primary progressive aphasia) and psychiatric conditions (depression, autism, addiction, schizophrenia, and attention disorders) with unequivocal efficacy (Lefaucheur, 2016). Studies of tDCS in DoC patients are presented in Table 3.

The first report of tDCS in DoC patients is from Angelakis et al. (2014), who showed an increase in CRS-R in 3 patients out of 10 with either a left DLPFC (L-DLPFC) or a left sensorimotor cortex repeated stimulation (5 sessions). However, this study was not controlled and the sham sessions were always performed before the repetitive sessions of active stimulation which doesn’t prevent a confound with spontaneous recovery. These encouraging results were further supported by a double-blind randomized controlled trial against sham published by Thibaut et al. (2014). In this study, the authors found a significant effect on consciousness of a single 2 mA L-DLPFC tDCS stimulation only in the MCS group, with an improvement in CRS-R in 13/30 (43%) MCS patients and 2/25 (8%) VS/UWS. Retrospective analysis of PET-TDM and MRI data of these patients prior stimulation yielded that tDCS responsiveness was characterized by preserved brain metabolism and gray matter integrity in cortical and subcortical regions traditionally involved in consciousness (prefrontal cortex, precuneus and thalamus) (Thibaut et al., 2015). Responders were also characterized by a higher connectivity in regions belonging to the extrinsic/executive control network in fMRI (Cavaliere et al., 2016) and increase theta connectivity and network centrality in EEG (Thibaut et al., 2018).

However, subsequent studies of single-session stimulation failed to reproduce the behavioral effect of tDCS (Naro et al., 2015a; Bai et al., 2016, 2017). Note though, that the stimulation parameters differed from those of the previous study, either due to smaller electrodes (25 cm^2^ vs. 35 cm^2^) or due to a distinct montage (orbitofrontal stimulation with anode between, Fp1 and Fp2 and cathode in Cz; Naro et al., 2015a). Yet these studies provided insight into the mechanisms of action of tDCS in DoC patients by combining the stimulation with other electrophysiological techniques (electroencephalogram -EEG-, event-related potentials -ERP- and/or transcranial magnetic stimulation -TMS). Hence, in a study combining TMS-EEG and tDCS over the L-DLPFC, Bai et al. (2017) showed that tDCS could modulate the cortical global excitability assessed by TMS with different spatial and temporal patterns in VS/UWS and MCS. In another study, the same authors showed that tDCS stimulation led to an increased fronto-parietal coherence in the theta band (Bai et al., 2016). Taken together, these results suggest that tDCS is able to modify the functional connectivity of consciousness-related networks as can be seen in healthy volunteers (Kunze et al., 2016) and could restore partially preserved long-range connectivity inside cortico-thalamic networks, thus explaining the better response rate observed in MCS patients.

**Table 3:** Transcranial direct current stimulation studies in DoC patients.

Study	Design/Control	Population	Stimulation parameters	Behavioral effect	Electrophysiological effect	Side effects
Angelakis et al., 2014	Prospective/Sham	10 patients (7 VS/UWS, 3 MCS)	5 sessions (20 min) of sham, 1 and 2 mA anodal L-DLPFC or L-SMC tDCS (F3/C3- Fp2; 25 cm2-35cm2)	CRS-R increase in the 3 MCS patients	Not assessed	None
Thibaut et al., 2014	Cross-over RCT/Sham	55 patients (25 VS/UWS, 30 MCS)	Single session (20 min) of 2 mA anodal L-DLPFC tDCS (F3-Fp2; 35 cm^2^)	Significant increase of CRS-R only in MCS patients.	Not assessed	None
Naro et al., 2015a	Cross-over RCT/Sham	25 patients (12VS/UWS, 10 MCS, 2 EMCS)	Single session (10 min) of 1 mA anodal orbito-frontal cortex (Fp-Cz; 25–35 cm^2^)	No effect	Changes in M1 excitability and premotor-motor connectivity in some DoC patients assessed by TMS	None
Naro et al., 2016b	Cross-over RCT/Sham	20 patients (10 VS/UWS and 10 MCS)	Single session (20 min) of 2 mA cerebellar 5 Hz oscillatory tDCS (medial cerebellum-left buccinator muscle; 16 cm^2^)	Improvement of CRS-R in MCS patients.	Increase in fronto-parietal coherence and power in theta and gamma band in MCS patients	None
Bai et al., 2017	Cross-over RCT/Sham	18 patients (9 VS/UWS, 9 MCS)	Single session (20 min) of 2 mA anodal L-DLPFC (F3-Fp2; 25 cm^2^)	No effect	Changes in cortical excitability assessed by TMS-EEG	Not reported
Bai et al., 2017	Cross-over RCT/Sham	17 patients (9 VS/UWS, 8 MCS)	Single session (20 min) of 2 mA anodal L-DLPFC (F3-Fp2; 25 cm^2^)	No effect	Increase fronto-parietal coherence in the theta band in MCS	Not reported
Zhang et al., 2017	Parallel RCT/Sham	26 patients (11 VS/UWS, 15 MCS)	20 sessions (20 min) of 2 mA anodal L-DLPFC (F3-Fp2; 35 cm2) over 10 consecutive days	Significant improvement in CRS-R in MCS patients	Increased P300 amplitude in MCS during an auditory oddball paradigm	None
Thibaut et al., 2017	Cross-over RCT/Sham	16 patients (all MCS)	5 sessions (20 min) of 2 mA anodal L-DLPFC (F3-Fp2; 35 cm^2^) over 5 days	Significant improvement of CRSR [in 9/16 (56%)] at 5 days, persisting at 12 days.	Not assessed	None
Huang w. et al., 2017	Cross-over RCT/Sham	27 patients (all MCS)	5 sessions (20 min) of 2 mA anodal posterior parietal cortex tDCS (Pz-Fp2; unknown)	Significant improvement of CRS-R after 5 days of stimulation, but no persistence at 10 days.	Not assessed	None
Estraneo et al., 2017	Cross-over RCT/Sham	13 patients (7 VS/UWS, 6 MCS)	5 sessions (20 min) of 2 mA anodal L-DLPFC F3-Fp2; 35 cm^2^) over 5 days	No effect on CRS-R after single or repeated sessions	Improvement of background rhythm in some patients	None
Martens et al., 2018	Cross-over RCT/Sham	27 patients (all MCS) in rehabilitation facilities or at home.	20 sessions (20 min) of 2 mA anodal L-DLPFC F3-Fp2; 35 cm^2^) over 4 weeks	No significant effect, but trend toward CRS-R improvement after 4 weeks, lasting at 12 weeks	Not assessed	One epileptic seizure

*Details of the montages are given as follow: target of the stimulation (electrodes positions according to the 10–20 international system; electrodes surface). CRS-R, Coma Recovery Scale – Revised; DoC, disorders of consciousness; MCS, Minimally Conscious State; EMCS, Emergence from Minimally Conscious State; L-DLPFC, left dorso-lateral prefrontal cortex; RCT, randomized controlled trial; SMC, sensory motor cortex; tDCS, transcranial direct current stimulation.*

In contrast to these single-session studies, in which the effect of tDCS appears transient, the repetition of tDCS sessions seems to increase both the rate and the amplitude of consciousness improvement. Indeed, Thibaut et al. (2017) showed in a double-blind cross-over randomized controlled trial, that repetitive sessions of L-DLPFC tDCS over five consecutive days led not only to an increased rate of response after the end of the stimulation period [significant improvement of CRS-R in 9 out of 16 (56%) MCS], but also that this improvement of consciousness was persisting 1 week after the last session of simulation. In another study, Zhang et al. (2017) further supported the efficacy of repetitive sessions (20 sessions in 10 consecutive working days) using a parallel controlled design coupling behavioral assessment with event-related potentials elicited during an auditory oddball paradigm. Together with a significant improvement of CRS-R scores, the authors reported an increased P300 amplitude, only after real stimulation in MCS (Zhang et al., 2017). It should, however, be noted that another study, despite similar design and stimulation parameters failed to show behavioral effects of both single-session and repetitive tDCS (Estraneo et al., 2017). These differences could be partially explained by a more heterogeneous population (inclusion of VS/UWS) farther away from the brain injury (more than a year in median). Interestingly, repetitive stimulation has also been tested in a home-based setting (home and rehabilitation facilities), in order to evaluate the feasibility of prolonged stimulation protocols by non-expert caregivers or family members (Martens et al., 2018). In this cross-over study by Martens et al. (2018), 27 chronic MCS received 4 weeks of tDCS and sham with a wash-out period of 8 weeks between the two. Overall adherence to treatment was very good (94%), but 5 patients received less than 80% of the planned sessions. This resulted in the absence of significant effect on CRS-R on the intention to treat analysis, but significant effect at the end of the stimulation and a trend at 8 weeks after the stimulation in the per protocol analysis.

While previous studies targeted the L- DLPFC, some authors tested other sites of stimulation. Naro et al. (2016a) reported that cerebellar stimulation, using 5-Hz oscillatory tDCS (otDCS), elicited an increase in fronto-parietal coherence and spectral power in the theta and gamma band in MCS patients, paralleled with CRS-R improvement. Repetitive stimulation of the posterior parietal cortex also resulted in a consciousness improvement but with a smaller and less prolonged effect that prefrontal cortex stimulation (Huang w. et al., 2017). Both these results show that tDCS is a reliable tool to modulate activity within widespread networks distant from stimulation sites. However, the major involvement of prefrontal cortex in cortico-subcortical networks and especially its dense connections the thalamus seems to make it the better target of stimulation in DoC.

Importantly, except for a single epileptic seizure, the aforementioned studies did not report major side effects, strengthening previous evidence that tDCS is safe (Matsumoto and Ugawa, 2017). This point is of utmost importance considering the frailty of this population.

#### Transcranial Alternative Current Stimulation (tACS)

In contrast to tDCS, tACS delivers a sinusoidal current through the scalp able to elicit entrain underlying oscillatory activity and synchronize large scale neuronal networks. Only one study reported the use of tACS in DoC patients (Naro et al., 2016b). In this double-blind randomized cross-over study, two sites of gamma range (35–140 Hz) tACS stimulation were tested (right DLPFC and frontopolar cortex), against an active transcranial random noise stimulation (tRNS) control condition. No changes in CRS-R score were observed, but all MCS and some VS/UWS showed increased in theta and gamma relative power and fronto-parietal coherence in response to DLPFC stimulation.

### Limitations and Perspectives of NIBS

While the therapeutic potential of rTMS in DoC patients seems limited so far, this review of the literature indicates a possible effect of tDCS in DoC patients. Indeed, several randomized controlled trials of tDCS in relatively large sample of DoC patients showed a significant behavioral improvement of consciousness, while rTMS studies failed to do so, maybe in part due to smaller sample sizes. Moreover, compared to rTMS, tDCS is together cheaper, less invasive, easier to use and more appropriate to repeated sessions, with consequently the potential of a wide availability for DoC patients, either during hospitalization or at home. However, please note that the current level of evidence is insufficient to issue recommendations on the use of both of these two techniques in DoC patients according to the latest guidelines on the therapeutic use of rTMS and tDCS (Lefaucheur et al., 2014, 2017) and further evidences from large-scale controlled studies are needed. Indeed, substantial heterogeneity remains to be explained and many factors are known to account for the variability of behavioral and electrophysiological effects of NIBS (Polanía et al., 2018).

Regarding tES, despite encouraging results, some authors still doubt the ability of weak intensity currents to elicit changes in brain activity. The group of Buszaki showed that with conventional intensities, electric fields barely reached the threshold for resting membrane potential modification in rodents and humans cadaver brains (Vöröslakos et al., 2018), but intracranial recordings in human epileptic patients showed current densities consistent with neurophysiological effects (Huang Y. et al., 2017). Nonetheless, higher intensities (up to 4 mA) could lead to better or more robust clinical effect while staying safe (Chhatbar et al., 2017). On the other hand, the ability of TMS to induce changes in cortical excitability is not debated, yet its use in DoC patients is not supported by current evidence and further studies are needed to demonstrate a potential benefit. In addition, safety and logistic concerns may harden its development in this condition.

While increasing the number of sessions of tDCS led to a better and more sustained response, in accordance with potential cumulative effect of induced synaptic plasticity, the optimal number sessions is still unknown as well as the safety of prolonged or intensive stimulation. Furthermore, these lasting changes are allegedly underpinned by NMDA mediation and tDCS efficacy is known to be reduced in the presence of ion-channel blockers (Wischnewski et al., 2018). Future studies should systematically report the use of such treatments to better explain individual response.

As for now, all studies of NIBS in DoC patients used standardized montage and sites of stimulation, irrespective of the individual anatomy of patients. Despite a low spatial resolution, this one-size-fits-all approach is probably misleading given the variability of lesions (etiology, locations, severity). Moreover, most studies quantifying and modeling electric fields were done in healthy subjects (Huang Y. et al., 2017; Ciechanski et al., 2018). Recently, MRI-based models of current distribution inside the brain have been developed for tDCS [SimNIBS (Saturnino et al., 2015), ROAST (Huang et al., 2018)]. In addition, coming studies should couple behavioral assessment with detailed functional imaging of the brain (EEG, fMRI, PET) before, during and after stimulation. First, imaging residual functional connectivity and brain metabolism before stimulation, which are seemingly major determinants of tDCS efficacy, as suggested by the better response rate observed in MCS patients, will help better select patients that could benefit from stimulation. Second, assessing the changes in those measures according to stimulation will allow to further understand the mechanism of consciousness improvement by NIBS. Finally, the combination of stimulation with functional imaging techniques will allow to probe the underlying brain activity of patients, which is known to considerably influence the neuromodulation properties of both for tES and TMS (Silvanto et al., 2008). In these non-communicative and fluctuating patients (Wannez et al., 2017), the continuous recording of brain activity could pave the way to the development of closed-loop stimulation protocol (Berényi et al., 2012; Ngo et al., 2013; Kozák and Berényi, 2017; Kozák et al., 2018) to foster conscious patterns of brain activity. Taken together, these tools presumably hold the promise to substantially optimize tES stimulation in DoC patients.

## Conclusion

Current evidence tends to support the efficacy of DBS and NIBS on consciousness in DoC patients (Thibaut and Schiff, 2018). However, while the latter is non-invasive and well tolerated, the former is associated with potential major side effects and should hence be reserved to selected patients. Less invasive techniques such as VNS are very promising and could represent a perfect trade-off between efficacy and invasiveness. Yet, evidence beyond the single-patient proof-of-concept study is needed to confirm its potential. Currently, we propose that all chronic DoC patients should be given the possibility to benefit from NIBS, and that tDCS should be preferred over rTMS given the evidence of the literature and its simpler use.

In any cases, future studies should systematically combine the stimulation with structural and functional brain-imaging, to (1) define patients who could benefit from the stimulation based on their residual brain activity (2) develop new stimulation protocols based on the understanding of the underlying mechanisms of consciousness improvement by electrical stimulation (3) tailor the stimulation to individual subjects based on their anatomy and/or functional brain-imaging through the use of computational modeling. This will also help define the relative place of each of these techniques in the treatment of DoC patients. One could imagine a progressive strategy, with a first-line use of NIBS to probe the possible response to stimulation followed by a second-line invasive stimulation to elicit sustained improvement of consciousness in carefully selected patients in which it is predicted to work. By then, some innovative and non-invasive stimulation techniques targeting deep brain structures, such as low intensity focused ultrasound pulsation (Monti et al., 2016), transcutaneous stimulation of the vagus nerve at the ear (Dietrich et al., 2008; Yu et al., 2017), or even indirect electrical brain stimulation through the olfactory receptors by using a nose-implanted electrode (Weiss et al., 2016) may turn to be efficient in DoC patients.

## Author Contributions

PB and BH reviewed the literature. PB and BH drafted the manuscript. All authors critically revised the manuscript for important intellectual content.

## Conflict of Interest Statement

The authors declare that the research was conducted in the absence of any commercial or financial relationships that could be construed as a potential conflict of interest.
